# Sacituzumab govitecan as second-line treatment for metastatic triple-negative breast cancer—phase 3 ASCENT study subanalysis

**DOI:** 10.1038/s41523-022-00439-5

**Published:** 2022-06-09

**Authors:** Lisa A. Carey, Delphine Loirat, Kevin Punie, Aditya Bardia, Véronique Diéras, Florence Dalenc, Jennifer R. Diamond, Christel Fontaine, Grace Wang, Hope S. Rugo, Sara A. Hurvitz, Kevin Kalinsky, Joyce O’Shaughnessy, Sibylle Loibl, Luca Gianni, Martine Piccart, Yanni Zhu, Rosemary Delaney, See Phan, Javier Cortés

**Affiliations:** 1 University of North Carolina Lineberger Comprehensive Cancer Center, Chapel Hill, NC USA; 2 Medical Oncology Department and D3i, Institut Curie, Paris, France; 3 Department of General Medical Oncology and Multidisciplinary Breast Centre, Leuven Cancer Institute, University Hospitals Leuven, Leuven, Belgium; 4 Department of Hematology/Oncology, Massachusetts General Hospital Cancer Center, Harvard Medical School, Boston, MA USA; 5 Department of Medical Oncology, Centre Eugène Marquis, Rennes, France; 6 Institut Claudius Regaud, IUCT-Oncopole, Toulouse, France; 7 Division of Medical Oncology, Department of Medicine, University of Colorado Anschutz Medical Campus, Aurora, CO USA; 8 Medical Oncology Department, Oncologisch Centrum, UZ Brussel, Brussels, Belgium; 9 Miami Cancer Institute, Miami, FL USA; 10 Department of Medicine, University of California San Francisco Helen Diller Family Comprehensive Cancer Center, San Francisco, CA USA; 11 Department of Medicine, Division of Hematology/Oncology, David Geffen School of Medicine, University of California, Los Angeles, Jonsson Comprehensive Cancer Center, Los Angeles, CA USA; 12 Winship Cancer Institute, Emory University, Atlanta, GA USA; 13 Baylor University Medical Center, Texas Oncology, US Oncology, Dallas, TX USA; 14 Department of Medicine and Research, Hämatologisch-Onkologische Gemeinschaftspraxis am Bethanien-Krankenhaus, Frankfurt, Germany; 15 Medical Oncology, Gianni Bonadonna Foundation, Milan, Italy; 16 Medical Oncology Department, Institut Jules Bordet and l’Université Libre de Bruxelles, Brussels, Belgium; 17 Department of Biostatistics, Gilead Sciences, Inc, Foster City, CA USA; 18 Department of Clinical Research, Gilead Sciences, Inc, Morris Plains, NJ USA; 19 Department of Clinical Development, Gilead Sciences Inc, Foster City, CA USA; 20 International Breast Cancer Center, Quirón Group, Barcelona, Universidad Europea de Madrid, Faculty of Biomedical and Health Sciences, Department of Medicine, Madrid, Vall d´Hebron Institute of Oncology (VHIO), Barcelona, Spain

**Keywords:** Cancer, Breast cancer

## Abstract

Patients with triple-negative breast cancer (TNBC) who relapse early after (neo)adjuvant chemotherapy have more aggressive disease. In the ASCENT trial, sacituzumab govitecan (SG), an antibody-drug conjugate composed of an anti-Trop–2 antibody coupled to SN-38 via a hydrolyzable linker, improved outcomes over single-agent chemotherapy of physician’s choice (TPC) in metastatic TNBC (mTNBC). Of 468 patients without known baseline brain metastases, 33/235 vs 32/233 patients (both 14%) in the SG vs TPC arms, respectively, received one line of therapy in the metastatic setting and experienced disease recurrence ≤12 months after (neo)adjuvant chemotherapy. SG prolonged progression-free survival (median 5.7 vs 1.5 months [HR, 0.41; 95% CI, 0.22–0.76]) and overall survival (median 10.9 vs 4.9 months [HR, 0.51; 95% CI, 0.28–0.91]) vs TPC, with a manageable safety profile in this subgroup consistent with the overall population. In this second-line setting, as with later-line therapy, SG improved survival over conventional chemotherapy for patients with mTNBC.

## Introduction

Metastatic triple-negative breast cancer (mTNBC) is a heterogeneous disease with few treatment options and poor outcomes^1–4^. This is largely due to the inherently aggressive clinical behavior of mTNBC and the lack of recognizable molecular targets for therapy^3,4^. Until recently, single-agent chemotherapy was the standard of care treatment option for previously treated mTNBC but it is associated with short progression-free survival (PFS), low response rates, and significant toxicity^5,6^. In the second-line (2L) or later mTNBC setting, single-agent chemotherapy results in a median PFS of <3 months and an objective response rate (ORR) of 11%^6–9^.

In addition to the poor response and survival outcomes with standard single-agent chemotherapy in the 2L mTNBC setting, patients with localized TNBC, despite treatment with neoadjuvant chemotherapy, have a higher risk of relapse and death within the first three years compared with patients with other types of breast cancer^10–12^. Patients with localized TNBC whose disease relapses following neoadjuvant chemotherapy also have significantly shorter survival than those with non-TNBC; this is likely due to the aggressive disease attributes of TNBC relative to other types of breast cancer and the lack of targeted therapies^10^. An additional negative prognostic feature is early relapse after diagnosis. Patients who relapse within 12 months of completing adjuvant or neoadjuvant ([neo]adjuvant) treatment either have primary resistance or early acquired resistance to cytotoxic chemotherapy; shorter disease-free intervals are associated with poorer prognosis for subsequent lines of therapy^13,14^. This means that patients with TNBC who relapse within 12 months of completing (neo)adjuvant chemotherapy are considered resistant to chemotherapy, have particularly poor prognoses, and need improved treatments.

Sacituzumab govitecan (SG) is an antibody-drug conjugate (ADC) composed of a humanized trophoblast cell-surface antigen-2 (Trop-2) antibody coupled to an SN-38 payload, the active metabolite of the topoisomerase 1 inhibitor irinotecan, via a proprietary, hydrolyzable linker^15^. SG is a Trop–2-directed ADC that is distinct due to an antibody component with high specificity for Trop-2, a high drug-to-antibody ratio (7.6:1), the fact that internalization and enzymatic cleavage of SG by tumor cells is not required for SN-38 liberation from the antibody, and its bystander effect in tumor microenviroment^15–19^.

The pivotal confirmatory phase 3 ASCENT study (NCT02574455) showed a significant survival improvement with SG over treatment of physician’s choice (TPC; eribulin, vinorelbine, gemcitabine, or capecitabine) in heavily pre-treated mTNBC, with a tolerable safety profile primarily consisting of hematologic toxicities and diarrhea. Specifically, median PFS (5.6 vs 1.7 months), median overall survival (OS; 12.1 vs 6.7 months), ORR (35% vs 5%), clinical benefit rate (CBR; 45% vs 9%), and median duration of response (DOR; 6.3 vs 3.6 months) were all improved with SG compared with TPC^20^. In April 2021, SG received U.S. Food and Drug Administration (FDA) approval for the treatment of adult patients with unresectable locally advanced or mTNBC who have received two or more prior systemic therapies, at least one of them for metastatic disease^21,22^.

However, given that the SG payload is a cytotoxic chemotherapy, it was not clear if patients with more chemotherapy-resistant diseases would benefit equally. In this exploratory subanalysis from the ASCENT study, we assessed the efficacy and safety of SG in the subgroup of patients without known brain metastases, but with relapse within 12 months of completing (neo)adjuvant chemotherapy, and who had received only one line of therapy for the metastatic disease before enrolling in the ASCENT study.

## Results

### Patients

Of 468 patients without known baseline brain metastases (BMNeg), 33/235 and 32/233 patients (both 14%) in the SG and TPC arms, respectively, received one line of therapy in the metastatic setting and experienced disease recurrence ≤12 months after (neo)adjuvant chemotherapy. All patients in this 2L subgroup were female, with a median age of 49 years (range, 30–80) for those who received SG and 51 years (range, 30–80) for those who received TPC (Table 1). The majority of patients in the SG and TPC arms had TNBC at initial breast cancer diagnosis (79% vs 84%), while 21% and 16% of patients had a different subtype of breast cancer at initial diagnosis, respectively. The median time to metastatic disease was 13.3 months (range, 0.2–41.7) in the SG arm and 13.2 months (range, 6.9–121.7) in the TPC arm. Three patients in the SG arm and none in the TPC arm had known germline breast cancer susceptibility gene 1 or 2 (*BRCA1*/*2*) mutations. Patients who received both neoadjuvant and adjuvant therapy were considered to have received one prior line of therapy, and the median number of prior anticancer regimens was two for both the SG and TPC arms, including prior (neo)adjuvant therapy and first-line metastatic regimens. The most common prior systemic therapies for patients in the SG vs TPC arms were cyclophosphamide (91% vs 97%), paclitaxel (85% vs 91%), and carboplatin (58% vs 84%); the majority of patients received prior anthracyclines in the form of doxorubicin (48% vs 50%) or epirubicin (39% vs 44%), and prior use of checkpoint inhibitors was low (9% vs 13%), respectively. The most common prior systemic therapies received in the early stage setting (either in the neoadjuvant, adjuvant, or locally advanced settings) in the SG vs TPC arms were cyclophosphamide (88% vs 97%), paclitaxel (79% vs 91%), and doxorubicin (45% vs 50%; Supplementary Table 1). The most common prior systemic therapies received in the 1L metastatic setting were carboplatin (39% vs 53%), gemcitabine (36% vs 44%), and capecitabine (18% vs 28%). The most common prior systemic therapies by early stage versus metastatic setting can be found in the Supplement (Supplementary Table 1).

**Table 1:** Patient demographics and baseline characteristics in the second-line subgroup of patients negative for brain metastases.

	SG (*n* = 33)	TPC (*n* = 32)
Female—no. (%)	33 (100)	32 (100)
Median age—y (range)	49 (30–80)	51 (30–80)
ECOG PS—no. (%)		
0	17 (52)	10 (31)
1	16 (48)	22 (69)
Race or ethnic group—no. (%)		
White	26 (79)	27 (84)
Black	3 (9)	3 (9)
Asian	3 (9)	1 (3)
Other	1 (3)	1 (3)
Median baseline creatinine clearance—mL/min (range)	115 (62–249)	115 (61–213)
Serum bilirubin at baseline—no. (%)		
Normal (≤ULN)	31 (94)	32 (100)
>1 to ≤1.5× ULN	2 (6)	0
>1.5× ULN	0	0
Initial diagnosis of TNBC^a^—no. (%)		
Yes	26 (79)	27 (84)
No	7 (21)	5 (16)
Median time to metastatic disease^b^—mo (range)	13.3 (0.2–41.7)	13.2 (6.9–121.7)
Median number of metastatic sites—no. (range)	2 (1–7)	3 (1–8)
Number of metastatic sites—no. (%)		
<3	17 (51.5)	12 (37.5)
≥3	16 (48.5)	20 (62.5)
Sites of metastatic disease^c^		
Lung	19 (58)	17 (53)
Liver	14 (42)	16 (50)
Bone	6 (18)	4 (13)
Germline *BRCA1/2* mutational status—no. (%)		
Negative	19 (58)	19 (59)
Positive	3 (9)	0
Unknown	11 (33)	13 (41)
Setting of prior systemic therapies—no. (%)		
Adjuvant	20 (61)	13 (41)
Neoadjuvant	27 (82)	29 (91)
Metastatic	33 (100)	32 (100)
Locally advanced disease	0	1 (3)
Previous use of PARP inhibitors^d,e^—no. (%)	2 (6)	0
Previous use of checkpoint inhibitors—no. (%)	3 (9)	4 (13)
Most common prior systemic therapies—no. (%)		
Cyclophosphamide	30 (91)	31 (97)
Paclitaxel	28 (85)	29 (91)
Carboplatin	19 (58)	27 (84)
Capecitabine	18 (55)	18 (56)
Doxorubicin^f^	16 (48)	16 (50)
Epirubicin^g^	13 (39)	14 (44)

Second-line patients were defined as those who received 1 line of therapy in the metastatic setting and recurred ≤12 months after (neo)adjuvant chemotherapy, prior to study enrollment.

*BRCA* breast cancer susceptibility gene, *ECOG PS* Eastern Cooperative Oncology Group performance score, *NA* not available, *PARP* poly adenosine diphosphate-ribose polymerase, *TNBC* triple-negative breast cancer, *ULN* upper limit of normal, *SG* sacituzumab govitecan, *TPC* treatment of physician’s choice.

^a^Patients in study either had TNBC at initial diagnosis or had hormone receptor-positive disease that converted to hormone-negative at time of study entry.

^b^Only patients with complete date of diagnosis available for time from diagnosis of early stage disease (stage I, II, and III) to metastatic disease (stage IV) were included in this analysis (26 and 29 patients in the SG and TPC arm, respectively).

^c^Based on an independent central review of target and non-target lesions. The sites listed are not all-inclusive.

^d^Prior PARP inhibitor use in the post-neoadjuvant setting only.

^e^PARP inhibitor received was olaparib in both patients (1 in the adjuvant setting; 1 in the metastatic setting).

^f^Includes doxorubicin and (liposomal) doxorubicin hydrochloride.

^g^Includes epirubicin and epirubicin hydrochloride.

While demographics and baseline characteristics were generally similar between treatment arms (Table 1), certain differences were observed. In the SG arm, a higher proportion of patients had an Eastern Cooperative Oncology Group performance score (ECOG PS) of 0 (52% vs 31%). More patients in the SG arm received prior therapy in the adjuvant setting (61% vs 41%) compared with those in the TPC arm and less frequently in the neoadjuvant setting (82% vs 91%), respectively. A lower proportion of patients in the SG vs TPC arms received prior platinum therapy (carboplatin, 58% vs 84%; cisplatin, 9% vs 0%, respectively).

At data cutoff, no patients in either arm for this subgroup remained on treatment (Supplementary Fig. 1). In the SG arm, all 33 patients discontinued treatment due to disease progression. In the TPC arm, the most common reason for treatment discontinuation was disease progression (25 patients, 78%); two patients each (6%) discontinued due to adverse events (AEs) and withdrawal of consent, and one patient each (3%) discontinued due to investigator decision, treatment delay of >3 weeks, and death. In the 2L subgroup, the median duration of therapy was 4.2 months (range, 0.0–13.9) in the SG arm and 1.2 months (range, 0.0–15.3) in the TPC arm.

### Efficacy outcomes

**Fig. 1:**
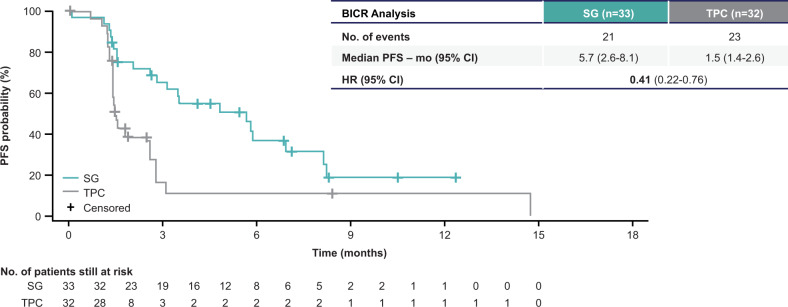
Progression-free survival in the second-line subgroup. Assessed by independent central review in the brain metastasis-negative population. Second-line patients were defined as those who received one line of therapy in the metastatic setting and recurred ≤12 months after (neo)adjuvant chemotherapy, prior to study enrollment. BICR blinded independent central review, HR hazard ratio, (neo)adjuvant neoadjuvant or adjuvant, PFS progression-free survival, SG sacituzumab govitecan, TPC treatment of physician’s choice.

**Fig. 2:**
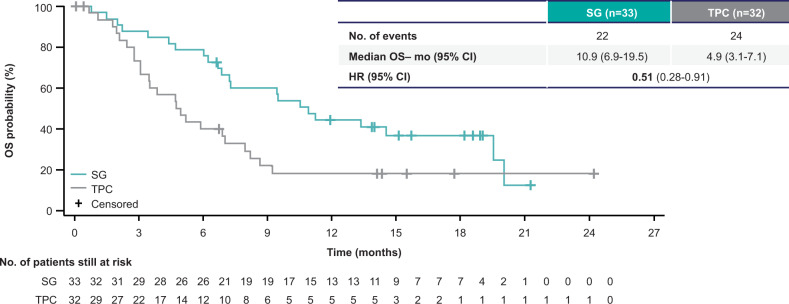
Overall survival in the second-line subgroup. Assessed in the brain metastasis-negative population. Second-line patients were defined as those who received one line of therapy in the metastatic setting and recurred ≤12 months after (neo)adjuvant chemotherapy, prior to study enrollment. HR hazard ratio, (neo)adjuvant neoadjuvant or adjuvant, OS overall survival, SG sacituzumab govitecan, TPC treatment of physician’s choice.

**Fig. 3:**
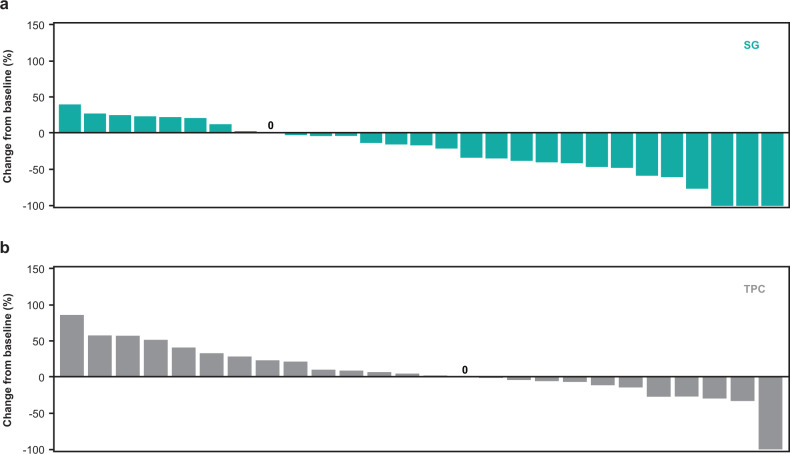
Best percent change in lesion size from baseline in the second-line subgroup. Waterfall plot showing best percent change from baseline in the sum of the diameters of target lesions in second-line patients treated with SG (**a**) or TPC (**b**). Assessed by independent central review in the brain metastasis-negative population. Second-line patients were defined as those who received 1 line of therapy in the metastatic setting and recurred ≤12 months after (neo)adjuvant chemotherapy, prior to study enrollment. SG sacituzumab govitecan, TPC treatment of physician’s choice.

Median PFS by central review was prolonged in the SG arm (5.7 months; 95% CI, 2.6–8.1) compared with TPC (1.5 months; 95% CI, 1.4–2.6) (hazard ratio [HR], 0.41; 95% CI, 0.22–0.76; Fig. 1). Similarly, median OS was longer, at 10.9 months (95% CI, 6.9–19.5) with SG vs 4.9 months (95% CI, 3.1–7.1) with TPC (HR, 0.51; 95% CI, 0.28–0.91; Fig. 2). The majority of patients who received SG experienced a reduction in tumor burden (Fig. 3a), while the majority of patients who received TPC did not (Fig. 3b). Patients treated with SG had a centrally assessed higher ORR than those treated with TPC (30% vs 3%; Supplementary Table 2). In those who received SG, one patient had a complete response (CR) and nine patients had a partial response (PR). In those who received TPC, no patient had a CR and one patient had a PR. The median DOR was 6.7 months (95% CI, 2.9-not evaluable [NE]) for the SG arm and NE for the TPC arm. The CBR was 42% (14/33 patients) vs 6% (2/32 patients) with SG vs TPC treatment.

### Safety outcomes

The safety population in this subgroup included 33 patients in the SG arm and 33 patients in the TPC arm. The most common treatment-related AEs (TRAEs) in the SG vs TPC arms were similar to the overall study population, including neutropenia (76% vs 24%), alopecia (70% vs 12%), diarrhea (58% vs 6%), nausea (52% vs 24%), anemia (39% vs 21%), and vomiting (36% vs 6%; Table 2). The most common grade ≥3 TRAEs in the SG vs TPC arms for this subgroup were neutropenia (61% vs 21%), leukopenia (9% vs 0%), diarrhea (6% vs 0%), anemia (3% vs 6%), and fatigue (3% vs 0%; Table 2). Grade ≥3 treatment-related neutropenia was seen in 61% of SG-treated patients, but febrile neutropenia in only one patient (grade 4); 58% of patients received concomitant granulocyte colony-stimulating factor (GCSF). Treatment-related diarrhea was primarily mild/moderate in severity, with few patients (6%) experiencing grade 3 events and none experiencing grade ≥4 events in the SG arm. There were no interstitial lung disease events in either arm. There was a low rate of discontinuation on the basis of treatment-emergent AE (6% in both SG and TPC arms), and no treatment-related deaths.

**Table 2:** Treatment-related adverse events (all grade, >20%; grade 3/4, >5% of patients) in the second-line subgroup.

	SG (*n* = 33)			TPC (*n* = 33)		
TRAE^a^—no. (%)	All grade	Grade 3	Grade 4	All grade	Grade 3	Grade 4
Hematologic						
Neutropenia^b^	25 (76)	13 (39)	7 (21)	8 (24)	5 (15)	2 (6)
Anemia^c^	13 (39)	1 (3)	0	7 (21)	2 (6)	0
Leukopenia^d^	5 (15)	3 (9)	0	2 (6)	0	0
Gastrointestinal						
Diarrhea	19 (58)	2 (6)	0	2 (6)	0	0
Nausea	17 (52)	0	0	8 (24)	0	0
Vomiting	12 (36)	0	0	2 (6)	0	0
Constipation	9 (27)	0	0	3 (9)	0	0
Other						
Alopecia	23 (70)	0	0	4 (12)	0	0
Fatigue	11 (33)	1 (3)	0	8 (24)	0	0

Assessed in the safety population of patients who received ≥1 dose of study treatment.

Second-line patients were defined as those who received one line of therapy in the metastatic setting and recurred ≤12 months after (neo)adjuvant chemotherapy, prior to study enrollment.

*AE* adverse event, *MedDRA* Medical Dictionary for Regulatory Activities, *NCI CTCAE* National Cancer Institute Common Terminology Criteria for AE, *SG* sacituzumab govitecan, *TPC* treatment of physician’s choice, *TRAE* treatment-related AE.

^a^Patients may report more than one event per preferred term. AEs were coded using MedDRA v22.1, and AE severity was graded per NCI CTCAE v4.03.

^b^Combined preferred terms of ‘neutropenia’ and ‘neutrophil count decreased’.

^c^Combined preferred terms of ‘anemia’, ‘hemoglobin decreased’, and ‘red blood cell count decreased’.

^d^Combined preferred terms of ‘leukopenia’ and ‘white blood cell count decreased’.

## Discussion

Due to limited treatment options beyond conventional chemotherapy and a poor prognosis, there is a great need for more effective treatments for mTNBC^23^. This exploratory, post-hoc analysis of the ASCENT trial assessed the efficacy and safety profile of SG in the 2L subgroup with particularly poor prognosis and evidence of primary chemorefractoriness. In the ASCENT trial, this subgroup represented 14% of the primary analysis population^20^. Treatment with SG demonstrated superior PFS (5.7 vs 1.5 months; HR, 0.41) and OS (10.9 vs 4.9 months; HR, 0.51) over the standard of care single-agent chemotherapy, with a manageable safety profile; Patients in the SG arm of this subgroup also received longer treatment and had durable benefit with SG treatment.

A few differences in baseline patient and disease characteristics across treatment arms were noted in this 2L subgroup analysis that may allude to differences in bulky or aggressive disease. Prior therapy in the adjuvant setting was more frequent in the SG vs TPC arm (61% vs 41%), though this does not appear to be a consequence of an earlier diagnosis of metastatic disease in the SG versus TPC arm (median, 13.3 vs 13.2 months). In addition, fewer patients in the SG vs TPC arm received prior platinum therapy (carboplatin, 58% vs 84%; cisplatin, 9% vs 0%, respectively), a trend consistent in both the early stage and metastatic settings; it is unclear why there was a large difference in prior platinum therapy use, particularly because it does not seem indicative of fewer patients with known germline *BRCA1/2* mutations in the SG vs TPC arm (9% vs 0%). Although patients in the TPC arm did have poorer functional status compared with patients in the SG arm (ECOG PS of 1, 69% vs 48%), no substantial differences in kidney and liver function between treatment arms were noted. Although patients in the SG arm had a lower median number of metastatic sites compared with the TPC arm (2 vs 3, respectively), organ involvement among the common sites of metastatic disease (e.g., lung, liver, bone) were similar between treatment arms. Taken together, there was no clear evidence of substantial differences in bulky or aggressive disease at baseline between the treatment arms that would require more active disease control.

The number of patients in this subgroup was limited to those whose disease relapsed within 12 months of completing (neo)adjuvant therapy; however, SG outperformed the physician’s choice 2L chemotherapy, suggesting that these patients derive clinical benefit from a cytotoxic-based therapy that is rationally delivered via a highly effective ADC. The results from this 2L subgroup analysis are consistent with that of the overall ASCENT trial population^20^. As the 2L subgroup in the present analysis represents a fraction of the total ASCENT study population, our results suggest that efficacy outcomes for SG in the third-line or higher mTNBC setting may be similar.

Single-agent chemotherapy is the usual treatment option for 2L mTNBC without germline pathogenic variants in *BRCA1* or *BRCA2*^5^. Patients who received single-agent taxane, gemcitabine, capecitabine, or vinorelbine in the 2L setting for mTNBC in the RIBBON-2 trial had a median PFS of 2.7 months, median OS of 12.6 months, and ORR of 18%^24^. Similarly, patients who received 2L single-agent eribulin in the recent retrospective TETRIS trial had a median PFS of 3.5 months and median OS of 11.9 months, similar to that observed with 2L or later eribulin in historical controls^9,25^. Patients who received single-agent chemotherapies in the 2L or later setting, including capecitabine, had similar survival outcomes, with a median PFS of <3 months and OS < 10 months^7,9,26^. Studies of single-agent checkpoint inhibitors have also shown low response rates and poor survival in 2L or later mTNBC^6,27^; in the KEYNOTE-119 trial, for example, pembrolizumab did not demonstrate an improvement in median OS over single-agent chemotherapy in the overall 2L or later mTNBC patient population (9.9 vs 10.8 months)^28^. The results seen in this ASCENT 2L subgroup analysis show that SG is a better option for these chemotherapy-resistant patients, as SG prolonged PFS by over 4 months, OS by 6 months, with a 10-fold higher response rate compared with conventional single-agent chemotherapy, even in this subpopulation selected for poor prognosis. These results supported the FDA approval of SG including the second-line setting, and suggest that SG efficacy is not affected by cross-resistance.

The safety profile of SG in this 2L subgroup was similar to that of the overall study population, with neutropenia, leukopenia, and diarrhea as the key TRAEs for SG. There did not appear to be an increased risk of neutropenia and diarrhea in these patients relative to the overall trial population^20^, and the majority of SG-related diarrhea events were of grade 1. Of note, peripheral neuropathy in 2L patients treated with SG was rare and of mild severity, with one grade 1 event (3%). Although the heterogeneity of the safety profiles associated with the individual agents in the TPC arm creates challenges in comparing adverse events among treatment arms in this small subpopulation, 2L patients who received TPC had higher rates of peripheral neuropathy (12%) and of a higher grade compared with those in the SG arm, likely attributable to the use of microtubule-directed drugs such as eribulin. Lastly, the frequency of treatment-related all-grade alopecia was higher in the SG vs TPC arm of the 2L subgroup (70% vs 12%). Although the mechanism behind this difference is unclear, a possible contributing factor may be that patients in the SG arm received treatment for a longer duration compared with those in the TPC arm (median of 4.2 vs 1.2 months, respectively).

In conclusion, the efficacy benefit and safety profile of SG in this 2L subgroup are consistent with that of the overall ASCENT study population across all key endpoints, suggesting that treatment with SG has improved efficacy outcomes over treatment with standard single-agent chemotherapy, with a manageable safety profile. Although SG is an ADC with a cytotoxic payload, these data suggest that SG may be able to overcome resistance to chemotherapy agents, including those used in earlier-stage settings for breast cancer. This subgroup analysis further supports that treatment with SG in the 2L mTNBC setting is as efficacious as in later lines and SG as a 2L treatment option for patients with mTNBC refractory to chemotherapy. Additional studies are ongoing (NeoSTAR, NCT04230109; SASCIA, NCT04595565) to further evaluate SG as an earlier-line treatment option for breast cancer.

## Methods

### Study design

ASCENT was an international, multicenter, randomized, phase 3 trial comparing the efficacy and safety of SG and TPC in patients with previously treated mTNBC. Details on the study have been previously published^20^. Briefly, patients received SG (10 mg/kg intravenously on days 1 and 8 of each 21-day cycle) or TPC (eribulin, vinorelbine, gemcitabine, or capecitabine) until disease progression or unacceptable toxicity. After discontinuation of study treatment, patients were followed every 4 weeks for survival, including documentation of any further therapy for their breast cancer. The primary endpoint was PFS (by blinded independent central review) as measured by computed tomography or magnetic resonance imaging per Response Evaluation Criteria in Solid Tumors (RECIST) version 1.1 in patients without known baseline brain metastases. Secondary endpoints included OS, ORR, DOR, and safety.

The ASCENT trial was conducted and approved by each investigational site institutional review board/ethics committee prior to initiation, and in accordance with the Declaration of Helsinki, International Council for Harmonisation Guidelines for Good Clinical Practice, FDA Code of Federal Regulations, national and local drug and data protection laws, and other applicable regulatory requirements. All patients provided written informed consent prior to enrollment.

### Subgroup patients

In the parent trial, patients were eligible if they had TNBC as defined by the standard American Society of Clinical Oncology/College of American Pathologists criteria^29,30^, and the disease was relapsed or refractory to two or more prior standard chemotherapy regimens (no upper limit) for unresectable, locally advanced or metastatic disease, and included a taxane (any setting). The primary analysis population was those without brain metastases. Per protocol, patients were eligible for ASCENT after only one prior regimen in the metastatic setting if their disease also recurred within 12 months of completing (neo)adjuvant therapy. These patients were eligible for this substudy. When both neoadjuvant and adjuvant therapy were given, this was considered to represent one line of therapy in this analysis.

### Statistical analysis

Efficacy outcomes for this post hoc subgroup analysis were assessed in the population of BMNeg patients who received one line of therapy in the metastatic setting and recurred within 12 months of completing (neo)adjuvant chemotherapy, prior to the study enrollment. Median PFS and ORR were assessed by blind independent central review per RECIST 1.1. Median PFS and median OS were assessed using Kaplan-Meier estimates, with HRs from unstratified Cox regression. Safety was assessed in the same population who received at least one dose of study treatment. AEs were coded using Medical Dictionary for Regulatory Activities (MedDRA) v22.1, and AE severity was graded per National Cancer Institute Common Terminology Criteria (NCI CTCAE) v4.0. Data cutoff was March 11, 2020.

### Reporting summary

Further information on research design is available in the Nature Research Reporting Summary linked to this article.

## Supplementary information

Supplemental Info

Reporting Summary

## Data Availability

Gilead Sciences shares anonymized individual patient data upon request or as required by law or regulation with qualified external researchers based on submitted curriculum vitae and reflecting nonconflict of interest. The requested proposal must also include a statistician. Approval of such requests is at Gilead Science’s discretion and is dependent on the nature of the request, the merit of the research proposed, the availability of the data, and the intended use of the data. Data requests should be sent to datarequest@gilead.com.

## References

[1] Kohler BA (2015). Annual report to the nation on the status of cancer, 1975–2011, featuring incidence of breast cancer subtypes by race/ethnicity, poverty, and state. J. Natl. Cancer Inst..

[2] Plasilova ML (2016). Features of triple-negative breast cancer: analysis of 38,813 cases from the national cancer database. Medicine.

[3] Newman LA, Reis-Filho JS, Morrow M, Carey LA, King TA (2015). The 2014 Society of Surgical Oncology Susan G. Komen for the Cure Symposium: triple-negative breast cancer. Ann. Surg. Oncol..

[4] Malorni L (2012). Clinical and biologic features of triple-negative breast cancers in a large cohort of patients with long-term follow-up. Breast Cancer Res. Treat..

[5] Cardoso F (2020). 5th ESO-ESMO international consensus guidelines for advanced breast cancer (ABC 5). Ann. Oncol..

[6] Li CH, Karantza V, Aktan G, Lala M (2019). Current treatment landscape for patients with locally recurrent inoperable or metastatic triple-negative breast cancer: a systematic literature review. Breast Cancer Res..

[7] Park IH (2019). Randomized open label phase III trial of irinotecan plus capecitabine versus capecitabine monotherapy in patients with metastatic breast cancer previously treated with anthracycline and taxane: PROCEED trial (KCSG BR 11-01). Cancer Res. Treat..

[8] Perez EA, Patel T, Moreno-Aspitia A (2010). Efficacy of ixabepilone in ER/PR/HER2-negative (triple-negative) breast cancer. Breast Cancer Res. Treat..

[9] Pivot X (2016). Pooled analyses of eribulin in metastatic breast cancer patients with at least one prior chemotherapy. Ann. Oncol..

[10] Liedtke C (2008). Response to neoadjuvant therapy and long-term survival in patients with triple-negative breast cancer. J. Clin. Oncol..

[11] Lin NU (2012). Clinicopathologic features, patterns of recurrence, and survival among women with triple-negative breast cancer in the National Comprehensive Cancer Network. Cancer.

[12] Adel NG (2021). Current treatment landscape and emerging therapies for metastatic triple-negative breast cancer. Am. J. Manag. Care.

[13] Melvin JC (2016). Progression of breast cancer following locoregional ipsilateral recurrence: importance of interval time. Br. J. Cancer.

[14] Yardley DA (2013). Drug resistance and the role of combination chemotherapy in improving patient outcomes. Int. J. Breast Cancer.

[15] Goldenberg DM, Cardillo TM, Govindan SV, Rossi EA, Sharkey RM (2015). Trop-2 is a novel target for solid cancer therapy with sacituzumab govitecan (IMMU-132), an antibody-drug conjugate (ADC). Oncotarget.

[16] Cardillo TM (2015). Sacituzumab Govitecan (IMMU-132), an anti-trop-2/SN-38 antibody-drug conjugate: characterization and efficacy in pancreatic, gastric, and other cancers. Bioconjug. Chem..

[17] Goldenberg DM, Sharkey RM (2020). Sacituzumab govitecan, a novel, third-generation, antibody-drug conjugate (ADC) for cancer therapy. Expert Opin. Biol. Ther..

[18] Nagayama A, Vidula N, Ellisen L, Bardia A (2020). Novel antibody-drug conjugates for triple negative breast cancer. Ther. Adv. Med. Oncol..

[19] Lopez S (2020). Preclinical activity of sacituzumab govitecan (IMMU-132) in uterine and ovarian carcinosarcomas. Oncotarget.

[20] Bardia A (2021). Sacituzumab govitecan in metastatic triple-negative breast cancer. N. Engl. J. Med..

[21] FDA grants regular approval to sacituzumab govitecan for triple-negative breast cancer. News Release. FDA. April 7, 2021. https://www.fda.gov/drugs/resources-information-approved-drugs/fda-grants-regular-approval-sacituzumab-govitecan-triple-negative-breast-cancer (2021).

[22] TRODELVY® (sacituzumab govitecan-hziy) [package insert]. (Immunomedics, Inc., 2021).

[23] Wahba HA, El-Hadaad HA (2015). Current approaches in treatment of triple-negative breast cancer. Cancer Biol. Med..

[24] Brufsky A (2012). Second-line bevacizumab-containing therapy in patients with triple-negative breast cancer: subgroup analysis of the RIBBON-2 trial. Breast Cancer Res. Treat..

[25] Krasniqi E (2021). Second-line eribulin in triple negative metastatic breast cancer patients. Multicentre retrospective study: the TETRIS trial. Int J. Med. Sci..

[26] Twelves C (2014). Efficacy of eribulin in women with metastatic breast cancer: a pooled analysis of two phase 3 studies. Breast Cancer Res. Treat..

[27] Adams S (2019). Pembrolizumab monotherapy for previously treated metastatic triple-negative breast cancer: cohort A of the phase II KEYNOTE-086 study. Ann. Oncol..

[28] Winer EP (2021). Pembrolizumab versus investigator-choice chemotherapy for metastatic triple-negative breast cancer (KEYNOTE-119): a randomised, open-label, phase 3 trial. Lancet Oncol..

[29] Hammond ME (2010). American Society of Clinical Oncology/College of American Pathologists guideline recommendations for immunohistochemical testing of estrogen and progesterone receptors in breast cancer. J. Clin. Oncol..

[30] Wolff AC (2013). Recommendations for human epidermal growth factor receptor 2 testing in breast cancer: American Society of Clinical Oncology/College of American Pathologists clinical practice guideline update. J. Clin. Oncol..

