# Supplementary material for: Sacituzumab govitecan as second-line treatment for metastatic triple-negative breast cancer—phase 3 ASCENT study subanalysis
Source: NPJ Breast Cancer. 2022 Jun 9;8:72. doi: 10.1038/s41523-022-00439-5 (PMC9184615; doi:10.1038/s41523-022-00439-5)
Supplement: Supplementary file 1 — Supplemental Info [file 41523_2022_439_MOESM1_ESM.docx]

**SUPPLEMENTARY INFORMATION**

**Title:** Sacituzumab govitecan as second-line treatment for metastatic triple-negative breast cancer—phase 3 ASCENT study subanalysis

Carey LA, et al.

**Supplementary Table 1. Most Common Prior Systemic Therapies by Setting in the Second-Line Subgroup**

| Most common prior  systemic therapies^a^—no. (%) | Early Stage Setting | | Metastatic Setting | |
| --- | --- | --- | --- | --- |
|  | SG (n=33) | TPC (n=32) | SG (n=33) | TPC (n=32) |
|  |  |  |  |  |
| Cyclophosphamide | 29 (88) | 31 (97) | 2 (6) | 1 (3) |
| Paclitaxel | 26 (79) | 29 (91) | 5 (15) | 0 |
| Carboplatin | 8 (24) | 11 (34) | 13 (39) | 17 (53) |
| Capecitabine | 13 (39) | 9 (28) | 6 (18) | 9 (28) |
| Doxorubicin^b^ | 15 (45) | 16 (50) | 1 (3) | 1 (3) |
| Epirubicin^c^ | 12 (36) | 14 (44) | 2 (6) | 1 (3) |
| Gemcitabine^d^ | 0 | 1 (3) | 12 (36) | 14 (44) |

Second-line patients were defined as those who received 1 line of therapy in the metastatic setting and recurred ≤12 months after (neo)adjuvant chemotherapy, prior to study enrollment.

^a^ Most common prior therapies when all settings are combined. Patients may have received an agent in both the early stage and metastatic setting; use in each setting was counted separately
^b^ Includes doxorubicin and (liposomal) doxorubicin hydrochloride.
^c^ Includes epirubicin and epirubicin hydrochloride.

^d^ Includes gemcitabine and gemcitabine hydrochloride.

SG, sacituzumab govitecan; TPC, treatment of physician’s choice.

**Supplementary Table 2. Treatment Responses in the Second-Line Subgroup of Patients Negative for Brain Metastases and With Relapse Within 12 Months of (Neo)adjuvant Treatment**

| BICR analysis | SG  (n = 33) | TPC  (n = 32) |
| --- | --- | --- |
| ORR—no. (%) | 10 (30) | 1 (3) |
| Best overall response—no. (%) |  |  |
| CR | 1 (3) | 0 |
| PR | 9 (27) | 1 (3) |
| SD | 13 (39) | 7 (22) |
| SD >6 months | 4 (12) | 1 (3) |
| PD | 9 (27) | 18 (56) |
| Not evaluable | 1 (3) | 6 (19) |
| CBR^a^—no. (%) | 14 (42) | 2 (6) |
| Median DOR—mo. (95% CI) | 6.7 (2.9-NE) | NE |

Second-line patients were defined as those who received one line of therapy in the metastatic setting and recurred ≤12 months after (neo)adjuvant chemotherapy, prior to study enrollment.

^a^ CBR is defined as the percentage of patients with a confirmed best overall response of CR or PR and SD ≥6 months.
BICR, blind independent central review; CBR, clinical benefit rate; CR, complete response; DOR, duration of response; NE, not evaluable; ORR, objective response rate; PD, progressive disease; PR, partial response; SD, stable disease; SG, sacituzumab govitecan; TPC, treatment of physician’s choice.

**Supplementary Figure 1. CONSORT Diagram for the Second-Line Subgroup of Patients Negative for Brain Metastases**

**
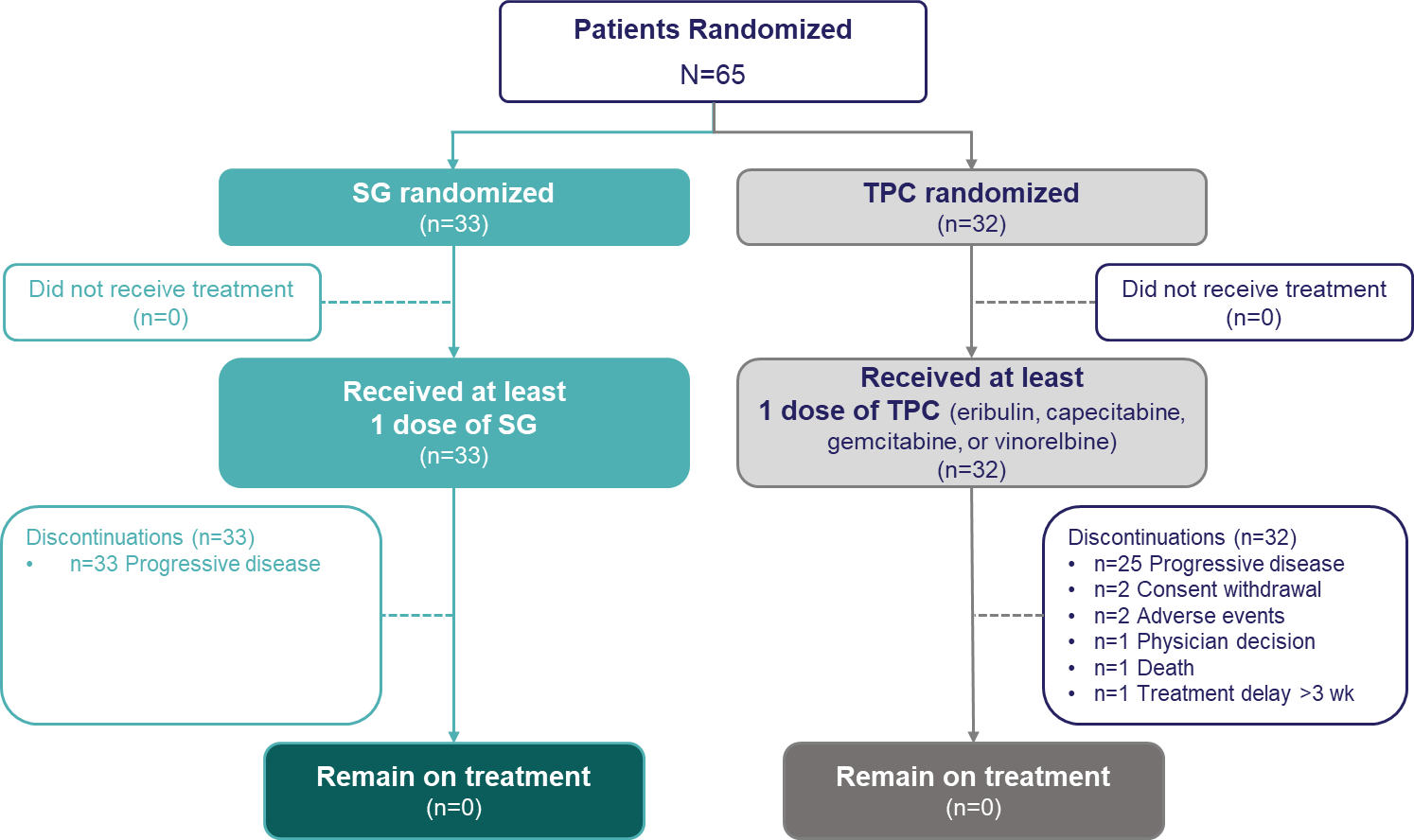
**

Second-line patients were defined as those who received one line of therapy in the metastatic setting and recurred ≤12 months after (neo)adjuvant chemotherapy, prior to study enrollment.

(Neo)adjuvant, neoadjuvant or adjuvant; SG, sacituzumab govitecan; TPC, treatment of physician’s choice.
