# Supplementary material for: Sacituzumab govitecan as second-line treatment for metastatic triple-negative breast cancer—phase 3 ASCENT study subanalysis
Source: NPJ Breast Cancer. 2022 Jun 9;8:72. doi: 10.1038/s41523-022-00439-5 (PMC9184615; doi:10.1038/s41523-022-00439-5)
Supplement: Supplementary file 2 — Reporting Summary [file 41523_2022_439_MOESM2_ESM.pdf]

nature portfolio

Corresponding author(s): Dr. Lisa A. Carey

Last updated by author(s): Jan 28, 2022

Reporting Summary

Nature Portfolio wishes to improve the reproducibility of the work that we publish. This form provides structure for consistency and transparency in reporting. For further information on Nature Portfolio policies, see our Editorial Policies and the Editorial Policy Checklist.

Statistics

For all statistical analyses, confirm that the following items are present in the figure legend, table legend, main text, or Methods section.

n/a	Confirmed	
☐	☒	The exact sample size (n) for each experimental group/condition, given as a discrete number and unit of measurement
☐	☒	A statement on whether measurements were taken from distinct samples or whether the same sample was measured repeatedly
☐	☒	The statistical test(s) used AND whether they are one- or two-sided<br>*Only common tests should be described solely by name; describe more complex techniques in the Methods section.*
☒	☐	A description of all covariates tested
☐	☒	A description of any assumptions or corrections, such as tests of normality and adjustment for multiple comparisons
☐	☒	A full description of the statistical parameters including central tendency (e.g. means) or other basic estimates (e.g. regression coefficient) AND variation (e.g. standard deviation) or associated estimates of uncertainty (e.g. confidence intervals)
☒	☐	For null hypothesis testing, the test statistic (e.g. F, t, r) with confidence intervals, effect sizes, degrees of freedom and P value noted<br>*Give P values as exact values whenever suitable.*
☒	☐	For Bayesian analysis, information on the choice of priors and Markov chain Monte Carlo settings
☒	☐	For hierarchical and complex designs, identification of the appropriate level for tests and full reporting of outcomes
☒	☐	Estimates of effect sizes (e.g. Cohen's d, Pearson's r), indicating how they were calculated

Our web collection on statistics for biologists contains articles on many of the points above.

Software and code

Policy information about availability of computer code

Data collection	No software was used
Data analysis	No software was used

For manuscripts utilizing custom algorithms or software that are central to the research but not yet described in published literature, software must be made available to editors and reviewers. We strongly encourage code deposition in a community repository (e.g. GitHub). See the Nature Portfolio guidelines for submitting code & software for further information.

Data

Policy information about availability of data

All manuscripts must include a data availability statement. This statement should provide the following information, where applicable:

- Accession codes, unique identifiers, or web links for publicly available datasets
- A description of any restrictions on data availability
- For clinical datasets or third party data, please ensure that the statement adheres to our policy

Gilead Sciences shares anonymized individual patient data upon request or as required by law or regulation with qualified external researchers based on submitted curriculum vitae and reflecting non conflict of interest. The request proposal must also include a statistician. Approval of such requests is at Gilead Science's discretion and is dependent on the nature of the request, the merit of the research proposed, the availability of the data, and the intended use of the data. Data requests should be sent to datarequest@gilead.com.

March 2021

Field-specific reporting

Please select the one below that is the best fit for your research. If you are not sure, read the appropriate sections before making your selection.

☒ Life sciences ☐ Behavioural & social sciences ☐ Ecological, evolutionary & environmental sciences

For a reference copy of the document with all sections, see nature.com/documents/nr-reporting-summary-flat.pdf

Life sciences study design

All studies must disclose on these points even when the disclosure is negative.

Sample size	This is a post hoc subgroup analysis based on clinical data from the Phase 3 ASCENT study. Patients included in this subgroup analysis were those without baseline brain metastases who received one line of therapy in the metastatic setting and recurred within 12 months of completing (neo)adjuvant chemotherapy, prior to ASCENT enrollment (33 patients in the sacituzumab govitecan arm and 32 patients in the chemotherapy arm). Anticipated enrollment in the overall ASCENT trial was 488 patients, including patients with or without brain metastases at baseline, with a 15% cap for patients with brain metastases. Under the assumption of a hazard ratio for disease progression or death (sacituzumab govitecan vs. chemotherapy) of 0.667, 315 events of progression or death would provide an estimated 95% power to detect a significant between-group difference in progression-free survival in the primary efficacy population; the expected progression-free survival with sacituzumab govitecan was 4.5 months, as compared with 3 months with chemotherapy of physician's choice.
Data exclusions	No data were excluded from the analysis.
Replication	This is a post hoc subgroup analysis based on clinical data from the Phase 3 ASCENT study, and data are not replicated at this time.
Randomization	This is a post hoc subgroup analysis based on clinical data from the Phase 3 ASCENT study. In the overall ASCENT trial, patients were randomly assigned in a 1:1 ratio to receive sacituzumab govitecan or single-agent chemotherapy as determined before randomization: eribulin, vinorelbine, capecitabine or gemcitabine. Patients were stratified at randomization according to the number of previous chemotherapy regimens for advanced disease (2 or 3 vs. >3), the presence of known brain metastases at baseline (yes vs. no), and geographic region (North America vs. rest of the world).
Blinding	Blinding was not relevant to this study as ASCENT was an open-label clinical trial.

Reporting for specific materials, systems and methods

We require information from authors about some types of materials, experimental systems and methods used in many studies. Here, indicate whether each material, system or method listed is relevant to your study. If you are not sure if a list item applies to your research, read the appropriate section before selecting a response.

Materials & experimental systems

n/a	Involved in the study	
☒	☐	Antibodies
☒	☐	Eukaryotic cell lines
☒	☐	Palaeontology and archaeology
☒	☐	Animals and other organisms
☐	☒	Human research participants
☐	☒	Clinical data
☒	☐	Dual use research of concern

Methods

n/a	Involved in the study	
☒	☐	ChIP-seq
☒	☐	Flow cytometry
☒	☐	MRI-based neuroimaging

Human research participants

Policy information about studies involving human research participants

Population characteristics	Of 468 patients without known baseline brain metastases (BMNeg), 33/235 and 32/233 patients (both 14%) in the sacituzumab govitecan (SG) and treatment of physician's choice (TPC) arms, respectively, received one line of therapy in the metastatic setting and experienced disease recurrence ≤12 months after (neo)adjuvant chemotherapy. All patients in this subgroup were female, with a median age of 49 years (range, 30-80) for those who received SG and 51 years (range, 30-80) for those who received TPC. The majority of patients in the SG and TPC arms had triple-negative breast cancer at initial breast cancer diagnosis (79% vs 84%), while 21% and 16% of patients had a different subtype of breast cancer at initial diagnosis, respectively. Median time to metastatic disease was 13.3 months (range, 0.2-41.7) in the SG arm and 13.2 months (range, 6.9-121.7) in the TPC arm. Three patients in the SG arm and none in the TPC arm had known germline breast cancer susceptibility gene 1 or 2 (BRCA1/2) mutations. Patients who received both neoadjuvant and adjuvant therapy were considered to have received one prior line of therapy, and the median number of prior anticancer regimens was two for both the SG and TPC arms, including prior (neo)adjuvant therapy and first-line metastatic regimens.

Recruitment	This is a post hoc subgroup analysis based on clinical data from the Phase 3 ASCENT study. The ASCENT study enrolled patients with metastatic triple-negative breast cancer that was relapsed or refractory to two or more previous standard chemotherapy regimens (no upper limit) for unresectable, locally advanced or metastatic disease; previous therapy had to include a taxane (for any indication). Patients had to have triple-negative breast cancer according to standard American Society of Clinical Oncology–College of American Pathologists criteria. Patients with stable brain metastases for at least 4 weeks before treatment were eligible for the trial but were excluded from the primary end-point analysis.
Ethics oversight	The ASCENT trial was conducted and approved by each investigational site institutional review board/ethics committee prior to initiation, and in accordance with the Declaration of Helsinki, International Council for Harmonisation Guidelines for Good Clinical Practice, FDA Code of Federal Regulations, national and local drug and data protection laws, and other applicable regulatory requirements.

Note that full information on the approval of the study protocol must also be provided in the manuscript.

Clinical data

Policy information about clinical studies

All manuscripts should comply with the ICMJE guidelines for publication of clinical research and a completed CONSORT checklist must be included with all submissions.

Clinical trial registration	NCT02574455
Study protocol	The redacted study protocol is available through the cited primary manuscript: Bardia A, et al. N Engl J Med. 2021;384:1529-1541.
Data collection	A total of 529 patients with triple-negative breast cancer were enrolled in the ASCENT trial between November 2017 and September 2019 at 88 sites in seven countries across North America and Europe. The date of data cutoff was March 11, 2020.
Outcomes	The primary endpoint in ASCENT was progression-free survival (PFS, by blinded independent central review) as measured by computed tomography or magnetic resonance imaging per Response Evaluation Criteria in Solid Tumors (RECIST) version 1.1 in patients without known baseline brain metastases. Secondary endpoints included overall survival (OS), objective response rate (ORR), duration of response (DOR), and safety. Median PFS and ORR were assessed by blind independent central review per RECIST 1.1. Adverse events (AE) were coded using Medical Dictionary for Regulatory Activities (MedDRA) v22.1, and AE severity was graded per National Cancer Institute Common Terminology Criteria (NCI CTCAE) v4.0.
